# Parvovirus B19 infection in Tunisian patients with sickle-cell anemia and acute erythroblastopenia

**DOI:** 10.1186/1471-2334-7-123

**Published:** 2007-10-25

**Authors:** Faouzi Regaya, Lassad Oussaief, Mohamed Bejaoui, Mongi Karoui, Mohamed Zili, Ridha Khelifa

**Affiliations:** 1 Blood Bank, Charles Nicolles Hospital, 1006 Tunis, Tunisia; 2 Viral and Molecular Tumor Diagnostics Unit, Habib Thameur Hospital, 1008 Tunis, Tunisia; 3 National Bone Marrow Transplantation Center, 1006 Tunis, Tunisia

## Abstract

**Background:**

Human parvovirus B19 is the etiologic agent of erythema infectiosum in children. It is also associated with other clinical manifestations in different target groups. Patients with chronic hemolytic anemia are at high risk of developing acute erythroblastopenia following infection by the virus. They usually become highly viremic and pose an increased risk of virus transmission. Close monitoring of such high risk groups is required for epidemiologic surveillance and disease prevention activities. Here we report a molecular epidemiological study on B19 virus infection in Tunisian patients with chronic hemolytic anemia.

**Methods:**

This study was conducted on 92 young chronic hemolytic anemia patients who attended the same ward at the National Bone Marrow Transplantation Center of Tunis and 46 controls from a different hospital. Screening for IgM and IgG anti-B19 antibodies was performed using commercially available enzyme immunoassays and B19 DNA was detected by nested PCR in the overlapping *VP1/VP2 *region. DNA was sequenced using dideoxy-terminator cycle sequencing technology.

**Results:**

Anti-parvovirus B19 IgG antibodies were detected in 26 of 46 sickle-cell anemia patients, 18 of 46 β-thalassemia and 7 of 46 controls. Anti-parvovirus B19 IgM antibodies were detected only in 4 of the sickle-cell anemia patients: two siblings and two unrelated who presented with acute erythroblastopenia at the time of blood collection for this study and had no history of past transfusion. B19 DNA was detected only in sera of these four patients and the corresponding 288 bp nested DNA amplicons were sequenced. The sequences obtained were all identical and phylogenetic analysis showed that they belonged to a new B19 virus strain of Genotype1.

**Conclusion:**

A new parvovirus B19 strain of genotype1 was detected in four Tunisian patients with sickle-cell anemia. Virus transmission appeared to be nosocomial and resulted in acute erythroblastopenia in the four patients. The possibility of independent transmission of this B19 variant to the patients is unlikely in light of the present epidemiological data. However this possibility cannot be ruled out because of the low genetic variability of the virus.

## Background

Human parvovirus B19 belongs to the Erythrovirus genus of the Parvoviridae family and is the etiologic agent of erythema infectiosum or fifth disease in children [1,2]. Infections with this virus are very common and can result in a wide range of clinical manifestations depending on the patient's immunological and hematological status. In immunocompetent individuals B19 infections can be asymptomatic or benign and may cause erythema infectiosum and arthropathy [3]. Immunodeficient subjects may become chronically infected [4,5]. During pregnancy, the virus can be transmitted in utero sometimes leading to fetal hydrops and fetal death [6,7].

Parvovirus B19 has a specific tropism for erythroïd progenitor cells and thus can cause a temporary infection of the bone marrow eventually leading to a transient arrest in erythropoiesis [8]. Patients with hematological disorders are at risk of severe clinical illness especially in chronic hemolytic anemia such as sickle cell disease [9,10], thalassemia [11] and hereditary spherocytosis [12]. In these diseases erythroïd progenitor cell formation is increased to compensate for red blood cell lysis and B19 infection can suppress erythropoïesis and induce acute erythroblastopenia often referred to as transient aplastic crisis [13]. The patients usually become highly viremic and pose an increased risk of virus transmission. Close monitoring of such high risk groups for this viral infection is, therefore, of great importance for epidemiologic surveillance and disease prevention.

Parvovirus B19 is a highly conserved virus; however, molecular epidemiological studies have shown the existence of three distinct genotypes modestly diverging from each other in sequence by about 10% while not showing any apparent differences in pathogenicity [14]. Genotype 1 is represented by the prototype B19 virus and is the most prevalent. The first B19 variant to be discovered was V9 [15] which represents the rare genotype 3. Genotype 2 is substantially more frequent with representatives such as A6 and LaLi [16,17].

Only two epidemiological studies on human parvovirus B19 infection in Tunisia have been reported. The first one [18] was a comparative study on blood donors from Tunisia and Belgium and the second [19] was carried out on Tunisian patients with chronic rheumatismal affections. Specific anti-B19 IgG was found in 65% of the blood donors and 80.7% of the patients with rheumatismal affections, whereas specific IgM was present in less than 2% of the blood donors and was not detected in the patients. No further epidemiological studies or molecular data on parvovirus genotypes and putative variants circulating in Tunisia were reported.

Here we report, for the first time, a molecular epidemiological study on Parvovirus B19 infection in Tunisian chronic hemolytic anemia patients.

## Methods

### Patients and sera

Sera used in this study were taken from 92 Tunisian patients with chronic hematological disorders: 46 sickle-cell anemia and 46 β-thalassemia. Forty six patients affected with non-hematological diseases (11 diabetes, 11 tonsillitis, 12 rhinitis, and 12 nephritic syndrome) were taken as controls. Patients of these groups were 2 to 19 years old with mean ages of 7.4 years for β-thalassemia, 9.2 years for sickle-cell anemia and 7.5 years for the control group. The chronic hemolytic anemia patients attended the same ward at the National Bone Marrow Transplantation Center of Tunis for treatment and follow up, and the control group was from the Charles Nicolles Hospital, Tunis. Written informed consent was obtained from all individuals included in this study or their parents. All the work was conducted in compliance with the Helsinki Declaration and approved by the National Ethics Committee of Tunisia.

### Detection of anti-Parvovirus B19 antibodies

Specific enzyme immunoassays (Biotrin International, France) were used to detect anti-Parvovirus IgG and IgM in sera according to the instructions of the manufacturer. The colorimetric reaction was read using the LP 200 microplate reader (Diagnostic Pasteur, France). Statistical significance of the serologic data was assessed by the Chi-square test for equality of distributions.

### Detection of viral DNA

All necessary precautions were taken to prevent cross contamination. DNA extraction, PCR reagent preparation, DNA amplification and gel electrophoresis were carried out in separate rooms using separate sets of micropipettes and sterile filter-tips. In addition, DNA extraction and PCR reagent handling were done in dedicated safety cabinets under sterile conditions.

Undiluted sera (200 μl) were digested with 2 μl of proteinase k (20 mg/ml) at 56°C for 1 h. Insoluble aggregates were removed by centrifugation at 13000 × g for 30 min. DNA was extracted using the QIAamp DNA Blood kit (Qiagen, Hilden, Germany) as described by the manufacturer.

Nested PCR for the detection of B19 DNA was performed using two primer pairs [20] selected in the overlapping region common to the minor (*VP1*) and major (*VP2*) capsid protein genes. The outer primers were: sense, 5'-CAAAAGCATGTGGAGTGAGG-3' (nt 3187–3206); anti-sense, 5'-CTACTAACATGCATAGGCGC-3' (nt 3584–3565). The inner primers were: sense, 5'-CCCAGAGCACCATTATAAGG-3' (nt 3271–3290); anti-sense, 5'-GTGCTGTCAGTAACCTGTAC-3' (nt 3558–3539). Product sizes were 398 bp and 288 bp for the first and second round of PCR, respectively.

Target DNA was amplified in a 50 μl reaction mixture containing 5 μl of sample DNA, 50 mM KCl, 10 mM Tris-HCl (pH = 8.3), 1.5 mM MgCl2, 200 μM of each deoxyribonucleoside triphosphate, 1.25 U of Taq DNA polymerase (Promega) and either 0.5 μM of each outer primer or 1.0 μM of each inner primer.

Each PCR run included a blank control containing water instead of target DNA, a negative control consisting of DNA extracted from healthy human leucocytes appropriately diluted in normal human serum, and a positive control; the latter was prepared from a first-round PCR product obtained previously (Regaya et al, 2003). Additional precautions were taken in handling this PCR product. First, it was maximally diluted in normal human serum to minimize the possibility of cross contamination while still allowing reproducible amplification. Second, this B19 amplicon was sequenced prior to use for future reference in case of suspected cross contamination.

Amplification reactions were performed with a thermocycler program consisting of 30 cycles of denaturation at 94°C for 1 minute, annealing for 2 minutes at either 55°C with the outer primers, or 57°C with the inner primers, and extension at 72°C for 3 minutes. A final 5-minute extension step was added after the last cycle. Two microliters of the product obtained in the first PCR were subjected to the second PCR using the inner primers.

For the detection of amplified DNA, five microliters of each product from the first or second PCR round were subjected to electrophoresis in 2% agarose gels in the presence of ethidium bromide and visualized under UV light. DNA bands of approximately 288 bp following the second round of PCR indicated positive results. The absence of such bands in the negative and blank controls indicated the specificity of the PCR and the absence of cross contamination. The sensitivity of this nested PCR in our hands was approximately 10^2 ^to 10^3 ^B19 genome copies per 5 μl.

### DNA sequence analysis

Nested PCR products of the *VP1 *gene were purified from excised agarose gel bands using QIAquick spin columns (Qiagen, Hilden, Germany). Sequencing of the purified DNA was performed using the Taq Dideoxy Terminator cycle sequencing kit and an ABI Prism 377 DNA sequencer (Applied Biosystems). Sequencing was first done with the forward inner primer and then repeated with the reverse inner primer for each sample. Sequences were analyzed with three programs included in BioEdit [21], ClustalW for multiple alignments, DNA Maximum Likelihood program with Molecular Clock (DNAMLK) to generate phylogenetic tree files, and finally TreeView [22] to read tree files and draw phylogenetic trees. The nucleotide sequence data from this study were deposited at GenBank under the following accession numbers: [GenBank: EF121420, GenBank: EF121421, GenBank: EF121422, GenBank: EF121423].

## Results

### Serological and clinical data

The serological data of the three groups tested are summarized in Table 1. Anti-parvovirus B19 IgG antibodies were detected in 26 (56.5%) of the sickle-cell anemia, 18 (39.1%) of the β-thalassemia and 7 (15.2%) of the control patients (Χ ^2 ^= 15.19, DF = 2, p ≤ 0.00050296). Anti-parvovirus B19 IgM antibodies were detected in only 4 (8.7%) patients of the sickle-cell anemia group. These patients will be referred to as patients 1 to 4. They were all males; two siblings (patients 1 and 2) and two unrelated (patients 3 and 4). Their ages were 18, 14, 7 and 19 years for patients 1, 2, 3, and 4, respectively. Only patients 1 and 2 were also positive for anti-B19 IgG (Table 2). All four patients were diagnosed with acute erythroblastopenia at the time of blood collection for this study and had no past transfusion history. They presented with fever, malaise, pallor and no cutaneous rash. Laboratory evaluation showed severe anemia and profound reticulocytopenia (Table 2). The four patients recovered progressively following red cell transfusion. All the IgG positive/IgM negative patients presented with no symptoms of acute erythroblastopenia.

**Table 1:** Serological data of the patients with chronic hematological disorders and control group

Patients		Positive for Anti B19 antibodies			
Group tested	Number	IgM		IgG	
		Number	%	Number	%
Sickle-cell anemia	46	4	8.7	26	56.5
β-thalassemia	46	0	0	18	39.1
Control	46	0	0	7	15.2

**Table 2:** Laboratory findings for the four parvovirus B19-infected sickle-cell anemia patients

Patient	Anti-B19		Hemoglobin pattern	Hemoglobin Concentration	Erythrocyte count	Reticulocyte count
	IgM	IgG		(g/dl)	(10^6^cells/μl)	(cells/μl)
1	+	+	S/β_0_	5.5	1.90	3880
2	+	+	S/β_0_	4.7	1.66	8300
3	+	-	S/S	4.3	1.57	7850
4	+	-	S/S	5.0	2.34	11700

### PCR results

**Figure 1:**
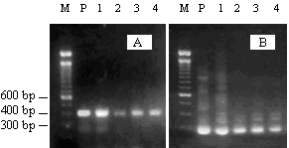
**Agarose gel electrophoresis pattern of parvovirus B19 DNA amplicons**. (A) 398 bp DNA bands obtained in the first round of PCR; (B) 288 bp DNA bands obtained in the second round of PCR. M: molecular weight marker (100 bp ladder, Invitrogen). P: positive control; lanes 1 to 4: amplicons of sera from patients 1 to 4, respectively.

Nested PCR in the overlapping *VP1/VP2 *region of the B19 genome was carried out on all anti-B19 antibody positive sera. Only sera from the four anti-B19 IgM-positive patients with acute erythroblastopenia produced DNA amplicons with electrophoretic bands of the size expected for B19 DNA, i.e. 398 bp in the first round (Figure 1A) and 288 bp in the second round (Figure 1B) of PCR. Such DNA bands were consistently absent from agarose gel lanes corresponding to the negative and blank controls throughout all our PCR work. These results showed the presence of parvovirus B19 DNA in only the sera of the four acutely infected IgM positive patients and indicated the absence of detectable chronic infection in the IgG positive/IgM negative subjects. The B19 virus isolates corresponding to the amplified DNA from the four acute erythroblastopenia patients were designated R1 to R4.

### Phylogenetic analysis

**Figure 2:**
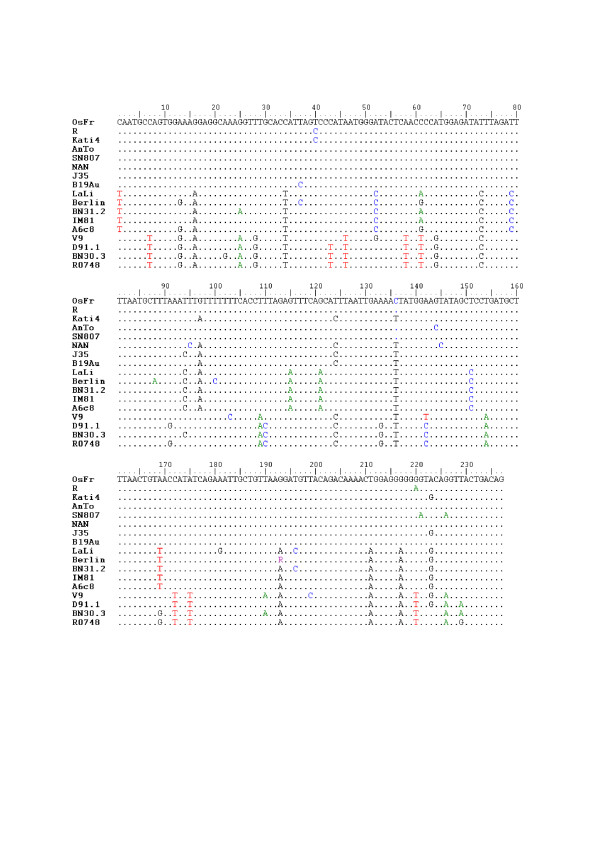
**Alignment of the partial DNA sequence of the four B19 isolates with reference strains**. The partial *VP1/VP2 *DNA sequence (R) representing the four B19 virus isolates R1 to R4 was aligned with representative sequences of the three human parvovirus genotypes retrieved from GenBank. Accession numbers were as follows. Genotype 1: OsFr [GenBank: DQ225150.1]; Kati 4 [GenBank: AF161226.1]; AnTo [GenBank: DQ225151.1]; SN807 [GenBank: DQ225149.1]; NAN [GenBank: AY504945.1]; J35 [GenBank: AY386330.1]; B19-Au [GenBank: M13178.1]. Genotype 2: LaLi [GenBank: AY044266.1]; Berlin [GenBank: AJ717293.1]; BN31.2 [GenBank: DQ333426.1]; IM-81 [GenBank: AY903437.1]; A6c8 [GenBank: AY064476.1]. Genotype 3: V9 [GenBank: AY345134.1]; D91.1 [GenBank: AY083234.1]; BN30.3 [GenBank: DQ408305.1]; R0748 [GenBank: DQ234779.1].

**Figure 3:**
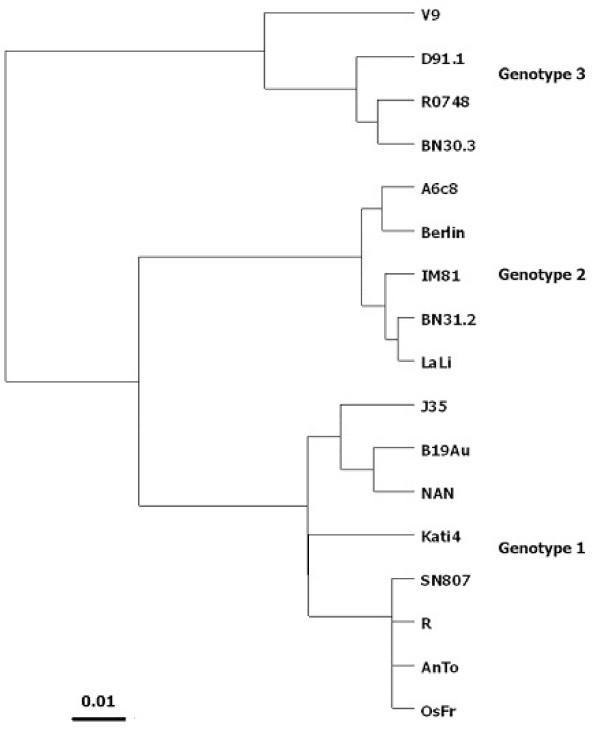
**Phylogenetic tree**. The phylogenetic tree of sequence R representing the four B19 virus isolates R1 to R4 was visualized by TreeView following alignment in BioEdit and Clustal W as shown in Figure 2. See legend to Figure 2 for accession numbers.

In order to identify the possible route of virus transmission to the four sickle-cell anemia patients and to know the genotype and sequence diversity of virus isolates R1, R2, R3, and R4, we purified and sequenced their nested DNA amplicons. Partial 237 nucleotide sequences were obtained from the sequencing data and aligned in BioEdit with ClustalW (not shown). The four sequences appeared to be identical, thus indicating that isolates R1 to R4 belonged to a single virus strain. The corresponding nucleotide sequence (R) was aligned in BioEdit with ClustalW against selected representatives of the three known B19 virus genotypes retrieved from GenBank (Figure 2) and a phylogenetic tree was constructed using DNAMLK and TreeView (Figure 3). Sequence R clustered with reference strains of genotype1. This sequence also produced many significant alignments in nucleotide BLAST (NCBI) and showed a maximum of 235/237 (99%) nucleotide identities with 11 reference strains of genotype 1 (not shown). A characteristic (T ↔ C) transition at position 40 was found exclusively in our isolates and in Kati 4 [GenBank: AF161226.1]. This transition is represented in the alignment of Figure 2. All these substitutions were silent as shown by alignment of sequence R in BLASTX (NCBI; not shown). Taken together, these data indicated that the virus isolates detected here represent a new B19 virus strain of genotype1.

## Discussion

Acute erythroblastopenia is the first disease to be associated with parvovirus B19 [9]. In predisposed patients such as those affected with chronic hemolytic anemia, 70 to 80% of erythroblastopenia cases are caused by infection with this virus. The infected patients usually become highly viremic and pose an increased risk of B19 virus transmission [11,13]. Close monitoring of such high risk groups is required to acquire data on parvovirus infection for the formulation of epidemiological surveillance programs and prevention strategies.

In this work we studied a population of 92 young Tunisian patients with chronic hemolytic anemia who attended the same ward at the National Bone Marrow Transplantation Center of Tunis. The control group consisted of patients with non-hematological disorders from different wards at a nearby hospital. The serological data obtained showed a much higher rate of past B19 infection in the chronic hemolytic anemia patients, as indicated by specific IgG. This situation may have resulted from a combination of known risk factors for B19 infection such as the presence of highly viremic patients, nosocomial transmission [23], and transfusion-related transmission [24].

Comparison between the present serological data and those previously reported in Tunisian blood donors [18] and patients with chronic rheumatismal disorders [19] illustrates the effect of age in the determinism of prevalence in B19 virus infection. The much older groups in the previous studies belonged to lower risk categories for B19 infection, yet they showed a significantly higher prevalence of past infection by the virus than the present high risk population. This observation corroborates published epidemiological reports indicating that this infection spreads at a high rate in low age groups to reach 50% of young adolescents by age 15, and continues at a lower rate throughout adult life to reach most of the elderly [25,26].

Four of the chronic hemolytic anemia patients were found to be acutely infected by the parvovirus as shown by the presence of specific IgM and viral DNA in their serum. The corresponding virus isolates had identical partial *VP1/VP2 *DNA sequences and therefore appeared to represent a single B19 strain, thus pointing towards nosocomial transmission. Since nosocomial transmission of B19 infection is documented [23,27] and since the patients had no past transfusions, the presence of the same virus strain in the four patients can be interpreted as the result of nosocomial transmission. An alternative explanation would be that the detected B19 strain is a hitherto unknown variant circulating independently of hospital settings. This possibility is also in conformity with the present genetic and phylogenetic analysis. Indeed, when the DNA sequence (R) representing this strain was aligned in nucleotide BLAST, it showed a maximum of 235/237 (99%) nucleotide identities with the closest reference strains, and a characteristic (T ↔ C) transition at nucleotide position 40 of R. This transition was also found in the Kati-4 strain which otherwise showed only 232/237 (97%) nucleotide identities with R. These findings indicated that the virus isolates represented a new B19 virus strain and phylogenetic analysis showed that it belongs to genotype1. Thus, the possibility that this strain is a newly identified circulating variant cannot be disregarded in view of the low variability of the *VP1/VP2 *gene [14,28]. However, it does not exclude the possibility of nosocomial transmission which is strongly suggested by the epidemiological data. Sequencing of a larger part of the genome of the present isolates, especially in the more variable *VP1 *unique region [27] and further detection and analysis of other isolates from Tunisia would be needed to confirm virus transmission and strain identity.

It is noteworthy, that all the nucleotide differences observed between this new strain and the reference strains of genotype1 in nucleotide BLAST were synonymous as shown by alignment of sequence R in BLASTX. This further confirmed the lower variability of the *VP1/VP2 *region at the protein level than at the DNA level [27].

## Conclusion

Four patients with sickle-cell anemia who presented with acute erythroblastopenia were found to be acutely infected by the B19 parvovirus. Analysis of the Partial viral DNA sequence in the overlapping *VP1/VP2 *region indicated the four patients were infected by a single B19 variant strain of genotype1. Virus transmission to the four patients was most probably nosocomial according to the epidemiological data. The molecular data were in line with this mode of transmission but could not fully confirm it because of the known low variability of the B19 genome especially in the *VP1/VP2 *gene. Final confirmation of transmission will await sequencing of longer and more variable regions of the viral genome.

## Competing interests

The author(s) declare that they have no competing interests.

## Authors' contributions

FR conceived the epidemiological studies, carried out the immunoassays and the PCR, participated in sequence alignments and drafted the manuscript. LO carried out DNA sequencing and participated in sequence analysis. MB established the diagnosis, collected the sera and compiled the clinical data. MK and MZ participated in the design of the study and its coordination. RK supervised the study, participated in its design, and finalized the manuscript. All authors read and approved the final manuscript.

## Pre-publication history

The pre-publication history for this paper can be accessed here:



## References

[B1] Young NS, Brown KE (2004). Parvovirus B19. N Engl J Med.

[B2] Anderson MJ, Jones SE, Fisher-Hoch SP, Lewis E, Hall SM, Bartlett CLR, Cohen BJ, Mortimer PP, Pereira MS (1983). Human parvovirus, the cause of erythema infectiosum (fifth disease)?. Lancet.

[B3] Plummer FA, Hammond GW, Forward K, Sekla L, Thompson LM, Jones SE, Kidd IM, Anderson MJ (1985). An erythema infectiosum-like illness caused by human Parvovirus infection. N Engl J Med.

[B4] Kurtzman GJ, Ozawa K, Cohen B, Hanson G, Oseas R, Young NS (1987). Chronic bone marrow failure due to persistent B19 parvovirus infection. N Engl J Med.

[B5] Kurtzman GJ, Cohen B, Meyers P, Amnullah A, Young NS (1988). Persistent B19 parvovirus infection as a cause of severe chronic anemia in children with acute lymphocytic leukemia. Lancet.

[B6] Brown T, Anand A, Ritchie LD, Clewley JP, Reid TMS (1984). Intrauterine parvovirus infection associated with hydrops fetalis. Lancet.

[B7] Levy R, Weissman A, Blomberg G, Hagay ZJ (1997). Infection byparvovirus B 19 during pregnancy: a review. Obstet Gynecol Surv.

[B8] Ozawa K, Kurtzman G, Young N (1986). Replication of the B19 parvovirus in human bone marrow cell cultures. Science.

[B9] Pattison JR, Jones SE, Hodgson J, Davis LR, White JM, Stroud CE, Murtaza L (1981). Parvovirus infections and hypoplastic crisis in sickle-cell anaemia. Lancet.

[B10] Serjeant GR, Serjeant BE, Thomas PW, Anderson MJ, Patou G, Pattison JR (1993). Human Parvovirus B19 infection in homozygous sickle cell disease. Lancet.

[B11] Lefrere JJ, Courouce AM, Bertrand Y, Girot R, Soulier JP (1986). Human parvovirus and aplastic crisis in chronic hemolytic anemias: a study of 24 observations. Am J Hematol.

[B12] Davidson RJ, Brown T, Wiseman D (1984). Human parvovirus infection and aplastic crisis in hereditary spherocytosis. J Infect.

[B13] Chorba T, Coccia P, Holman RC, Tattersall P, Anderson LJ, Sudman J, Young NS, Kurczynski E, Saarinen UM, Moir R (1986). The role ofparvovirus B19 in aplastic crisis and erythema infectiosum (fifth disease). J Infect Dis.

[B14] Servant A, Laperche S, Lallemand F, Marinho V, De Saint Maur G, Meritet JF, Garbarg-Chenon A (2002). Genetic diversity within human erythroviruses: identification of three genotypes. J Virol.

[B15] Nguyen QT, Sifer C, Schneider V, Allaume X, Servant A, Bernaudin F, Auguste V, Garbarg-Chenon A (1999). Novel human erythrovirusassociated with transient aplastic anemia. J Clin Microbiol.

[B16] Nguyen QT, Wong S, Heegaard ED, Brown KE (2002). Identificationand characterization of a second novel erythrovirus variant, A6. Virology.

[B17] Hokynar K, Soderlund-Venermo M, Pesonen M, Ranki A, Kiviluoto O, Partio EK, Hedman K (2002). A new Parvovirus genotype persistent inhuman skin. Virology.

[B18] Letaief M, Vanham G, Boukef K, Yacoub S, Muylle L, Mertens G (1997). Higher prevalence of parvovirus B19 in Belgian as compared toTunisian blood donors: differential implications for prevention of transfusional transmission. Transfus Sci.

[B19] Regaya F, Khelifa R, Zouari R, Kchir M, Karoui M, Essid R (2003). Research on Parvovirus B19 infections and chronic articular manifestations in a Tunisian hospital. Arch Inst Pasteur Tunis.

[B20] Yamakawa Y, Oka H, Hori S, Arai T, Izumi R (1995). Detection of human parvovirus B19 DNA by nested polymerase chain reaction. Obstetrics & Gynecology.

[B21] Hall TA BioEdit: biological sequence alignment editorfor Windows 95/98/NT version 5.0.7. http://www.mbio.ncsu.edu/BioEdit/bioedit.html.

[B22] Page RDM (1996). TreeView: an application to displayphylogenetic trees on personal computers. Computer Applications in the Biosciences.

[B23] Bell LM, Naides SJ, Stoffman P, Hodinka RL, Plotkin SA (1989). Human parvovirus B19 infection among hospital staff members after contact with infected patients. N Engl J Med.

[B24] Azzi A, Morfini M, Mannucci PM (1999). The transfusion-associated transmission of parvovirus B19. Transfus Med Rev.

[B25] (1989). Risks associated with human parvovirus B19 infection. MMWR Morb Mortal Wkly Rep.

[B26] (1987). Human parvovirus B19 infections in United Kingdom 1984–86. Lancet.

[B27] Takahashi N, Takada N, Hashimoto T, Okamoto T (1999). Genetic heterogeneity of the immunogenic viral capsid protein region of human parvovirus B19 isolates obtained from an outbreak in a pediatric ward. FEBS Letters.

[B28] Erdman DD, Durigon EL, Wang QY, Anderson LJ (1996). Genetic diversity of human parvovirus B19: sequence analysis of the VP1/VP2 gene from multiple isolates. J Gen Virol.

